# Glucocorticoid therapy and risk of bladder cancer

**DOI:** 10.1038/sj.bjc.6605314

**Published:** 2009-09-22

**Authors:** K Dietrich, A Schned, J Fortuny, J Heaney, C Marsit, K T Kelsey, M R Karagas

**Affiliations:** 1 Department of Community and Family Medicine, Section of Biostatistics and Epidemiology, Dartmouth Medical School, Lebanon, NH 03756, USA; 2 Department of Pathology, Dartmouth Medical School, Lebanon, NH 03756, USA; 3 Novartis Farmaceutica S.A., Gran Via de les Corts Catalanes 764, Barcelona E-08013, Spain; 4 Department of Surgery, Dartmouth Medical School, Lebanon, NH 03756, USA; 5 Department of Pathology, Brown University, Providence, RI 02912, USA; 6 Department of Community Health and Laboratory Medicine, Brown University, Providence, RI 02912, USA

**Keywords:** bladder cancer, transitional cell urothelial carcinoma, glucocorticoids, immunosuppressive therapy, case–control study

## Abstract

**Background:**

Use of immunosuppressive drugs post organ transplantation, and prolonged use of glucorticoids for other conditions have been associated with subsequent risk of certain malignancies, that is, skin cancers and lymphoma. There is evidence that the incidence of bladder cancer is also elevated among organ transplant recipients, however, it is unknown whether other groups of patients, that is, those taking oral glucocorticoids, likewise are at an increased risk.

**Methods:**

In a population-based case–control study in New Hampshire, USA, we compared the use of glucocorticoids in 786 bladder cancer cases and in 1083 controls. We used unconditional logistic regression analysis to compute adjusted odds ratios (ORs) associated with oral glucocorticoid use.

**Results:**

In our analysis, the risk of bladder cancer was related to a history of prolonged oral glucocorticoid use (OR=1.85, 95% CI=1.24–2.76, adjusted for age, gender and smoking). Associations with oral glucocorticoid use were stronger for invasive tumours (OR=2.12, 95% CI=1.17–3.85) and tumours with high (3+) p53 staining intensity (OR=2.35, 95% CI=1.26–4.36).

**Conclusion:**

Our results raise the possibility of an increased risk of bladder cancer from systemic use of glucocorticoids, and a potential role of immune surveillance in bladder cancer aetiology.

With an estimated 68 810 new cases in 2008, bladder cancer is the fifth most commonly diagnosed cancer in the United States of America (Scosyrev *et al*, 2009). Several risk factors have been identified as potential causes of bladder cancer, including tobacco use, occupational exposure to aromatic amines and polycyclic aromatic hydrocarbons (Johansson and Cohen, 1997; Silverman *et al*, 2006), and treatment with cyclophosphamide (Kinlen, 1985; Baker *et al*, 1987; Radis *et al*, 1995; Volkmer *et al*, 2005). Glucocorticoids, often in combination with other immunosuppressive drugs, are a part of post-transplant therapy and alone are used to treat other acute or chronic inflammatory conditions, including rheumatoid arthritis, inflammatory bowel disease and asthma (Zoorob and Cender, 1998). There is evidence of an enhanced risk of certain types of cancer, including skin cancers and lymphomas, among organ transplant recipients (Birkeland *et al*, 1995, 2000; Kyllonen *et al*, 2000; Adami *et al*, 2003; Vajdic *et al*, 2006) and prolonged users of immunosuppressive drugs, such as glucorticoids, for other conditions (Karagas *et al*, 2001; Sorensen *et al*, 2004; Jensen *et al*, 2009). The incidence of bladder cancer is two- to four-fold higher among organ transplant recipients (Buzzeo *et al*, 1997); however, the risk among patients using oral glucocorticoids for other reasons is unknown. Therefore, we examined the potential risk of bladder cancer associated with glucocorticoid use in non-transplant recipients as part of an ongoing population-based case–control study of bladder cancer conducted in New Hampshire, USA.

## Materials and methods

### Study group

Through the New Hampshire State Department of Health and Human Services’ rapid reporting Cancer Registry, we identified newly diagnosed cases of bladder cancer among New Hampshire residents, aged 25–74 during the 1 July 1994 to 31 December 2001 period. For efficiency, we shared controls with a study of non-melanoma skin cancer covering a diagnostic period of 1 July 1993 to 30 March 2000, along with additional controls frequency matched to the bladder cancer cases by age and gender (Wallace *et al*, 2009). Controls <65 years of age were selected using population lists obtained from the New Hampshire Department of Transportation. Controls 65 years of age and older were chosen from data files provided by the Centers for Medicare & Medicaid Services (CMS) of New Hampshire, as described previously (Fortuny *et al*, 2007).

### Interviews

We conducted standardized personal interviews with the study participants to obtain information on demographic traits, use of tobacco (including frequency, duration and intensity of cigarette smoking), alcohol and other exposures. We requested the original paraffin-embedded tumour specimen for histopathology re-review by the study pathologist who classified tumours according to WHO 1973 and WHO ISUP criteria (Schned *et al*, 2007). Owing to the high concordance rates for overall diagnosis (>90%), we classified subjects based on the original pathologist's diagnosis; whereas tumour morphology, extent of disease and grade were based solely on the standardized histopathology re-review by the study pathologist. Immunohistochemical analysis of the tumours was carried out for TP53 using a monoclonal antibody (BioGenex, San Ramon, CA, USA), and scored for intensity and the percentage of tumour cells staining positively as markers of tumour severity, as the number of tumours having TP53 mutations increases with the degree of invasiveness of the tumour (Kelsey *et al*, 2004) and may represent an aetiologically distinct subgroup of tumours (Kelsey *et al*, 2005; Wallace *et al*, 2009). We obtained informed consent from each participant and all procedures and study materials were approved by the Committee for the Protection of Human Subjects at Dartmouth College.

### Drug use assessment

Participants were asked if their doctor had ever prescribed glucocorticoids or steroids as pills, injections or inhalers for 1 month or longer before their reference date (defined as the diagnosis date of the cases and a comparable date randomly assigned to the controls). Those who responded positively were considered users and were asked the age they were first treated, the condition for which the glucocorticoids were prescribed, the name of the drug, dose and duration of the treatment. Those who responded that they did not use glucocorticoids for at least 1 month were considered non-users. To aid recall, we developed a list of glucocorticoids and other immunosuppressive drugs (trade name, generic name and description) and a pictorial guide showing the most commonly used drugs grouped by pill colour, size and shape. To minimize potential reporting bias, we did not reveal the specific hypotheses-of-interest to either the interviewer or participant, and we did not inform the interviewers of the case–control status of participants.

### Statistical analysis

We computed odds ratios (ORs) and their 95% confidence intervals (CIs) for bladder cancer associated with the use of glucocorticoids before the reference date using unconditional logistic regression, taking into account multiple confounding factors (Breslow and Day, 1980). In addition to age and sex, we also adjusted for the potential confounding effects of smoking status (current, former and never). Further, we assessed the possibility that education, as a marker of socioeconomic status, could act as a potential confounder, but the inclusion of this variable did not significantly influence our results, and therefore was not included in the final models.

We conducted a combined analysis of all bladder cancers, as well as specifically on pathologically confirmed transitional cell carcinomas. In addition, we carried out a case-only analysis comparing glucocorticoid users to non-users according to subgroups defined by the extent of disease (low grade non-invasive, high grade non-invasive, invasive or carcinoma *in situ*) and TP53 staining intensity (<3 or 3+) to examine whether glucocorticoid use was associated with tumour aggressiveness.

## Results

A total of 824 cases took part in the study (85% response rate of those eligible), and from 786 (95%) we obtained data on the use of oral glucocorticoids. A total of 1119 controls took part in the study (70% of those eligible), and from 1083 (97%) we obtained data on the use of oral glucocorticoids (Table 1). Cases were more likely male than were controls, but did not differ by age. A higher percentage of cases than controls were current smokers, who reported a family history of bladder cancer and did not have education beyond high school or technical college (Table 1). Overall, 5% of controls and 8% of cases reported a history of oral glucocorticoid use for 1 month or longer. Prednisone accounted for 87% of the reported oral glucocorticoid use. Of the histologically reviewed cancers, 98% were transitional cell (urothelial) carcinomas (TCC), 61% were non-invasive low grade, 8% were non-invasive high grade, 27% were invasive and 4% were *in situ* carcinomas (Table 1).

### Associations with glucocorticoid use

Any glucocorticoid use was associated with an increased risk of bladder cancer, and the association was stronger for oral use (OR=1.85, 95% CI=1.24–2.76) than inhaled use (Table 2). Although there was not a clear trend in risk by categories of duration of use, the OR was highest among those who used oral glucocorticoids for 5 years or longer (OR=3.39, 95% CI=0.98–11.74) (Table 2). Data on dose was available on 63 (65%) of the 97 individuals who reported prednisone use. Examining maximum daily reported dose, we detected an elevated OR primarily in the strata of ⩾50 mg (OR=4.12, 95% CI=1.12–15.15), but not for <10 mg (OR=0.76, 95% CI=0.25–2.34) or for 10–49 mg (OR=1.07, 95% CI=0.43–2.70). Stratifying by reasons for use led to small strata, but did not suggest confounding by indication (Table 2). ORs appeared higher among never smokers (OR=5.24, 95% CI=2.20–12.50) than smokers (OR=1.42, 95% CI=0.91–2.21) (*P* for interaction=0.004); however, ORs were elevated in both groups and there were few never smokers.

### Subgroups by tumour characteristics

Restriction to transitional cell carcinomas did not affect our risk estimate for oral glucocorticoid use (TCC OR=1.85, 95% CI=1.22–2.81). Further, using a case–case approach, we found higher ORs associated with more advanced disease stage and grade (Table 3). Oral glucocorticoid users had somewhat higher odds of having TP53 high-intensity stained tumours than TP53 negative tumours.

## Discussion

Immunosuppressive therapy, usually a combination of cytotoxic drugs and glucocorticoids, is commonly prescribed to organ transplant recipients, in order to prevent allograft rejection. Glucocorticoids, alone or in combination with other immunosuppressive drugs, are used to help treat many chronic inflammatory conditions, such as rheumatoid arthritis and asthma. Long-term immunosuppressive therapy has been shown to increase the risk of many types of malignancies among organ transplant recipients (Birkeland *et al*, 1995, 2000; Kyllonen *et al*, 2000; Adami *et al*, 2003; Vajdic *et al*, 2006). Further, previous research has found increased risk of skin cancers and non-Hodgkin's lymphoma among long-term glucocorticoid users (Karagas *et al*, 2001; Sorensen *et al*, 2004; Jensen *et al*, 2009). Our findings suggest that long-term glucocorticoid users may have an increased risk of bladder cancer, although to a lesser extent than organ transplant recipients.

Previous large cohort studies have found risk of bladder cancer in transplant recipients to be elevated two- to five-fold over the general population, a smaller effect size than seen with skin cancer or lymphoma (Kyllonen *et al*, 1994; Buzzeo *et al*, 1997; Adami *et al*, 2003). Bladder cancer is a far less common disease than skin cancer, and thus it is not surprising that cohort studies would observe few if any cases (Master *et al*, 2004; Kamal *et al*, 2007). An exception was a Taiwanese study that reported bladder cancer as the most common type of post-transplant malignancy with an incidence of 4.1% among kidney transplant recipients (Wu *et al*, 2004).

In non-transplant cohorts, cyclophosphamide and other drugs with immunosuppressive effects also have been linked to increased risk of bladder cancer. Several previous studies have found that cyclophosphamide therapy in patients with Wegener’s granulomatosis is associated with an increased risk of bladder cancer (Travis *et al*, 1995; Talar-Williams *et al*, 1996; Knight *et al*, 2004). Patients with rheumatoid arthritis treated with long-term cyclophosphamide (Radis *et al*, 1995) and patients on cyclophosphamide or azathioprine for underlying autoimmune disorders (e.g., rheumatoid arthritis, Crohn’s disease) had reported increased risks of bladder cancer (Kinlen, 1985). In another study, patients with rheumatic disease on multiple drugs, including azathioprine, methotrexate, cyclophosphamide and chlorambucil were found to have an enhanced risk of neoplasms, including in the bladder (Asten *et al*, 1999). In a more recent cohort study, rheumatoid arthritis patients on either methotrexate or tumour necrosis factor alpha (TNF-*α*) inhibitors, were twice as likely to develop bladder cancer as the general population (Setoguchi *et al*, 2006).

An enhanced risk of bladder cancer also has been observed in other immunosuppressed populations, including HIV-infected and AIDS patients. Manfredi *et al* (2006), published a case series and literature review with thirteen reports of HIV-infected patients with bladder carcinoma. Although the prevalence of bladder cancer is less than Kaposi’s sarcoma, lymphoma and cervical cancer, this report is consistent with our observations and suggests the necessity of further research addressing the association of bladder cancer with immunosuppression. Further in recent work, Roberts *et al* (2008) found evidences of polyoma virus in urothelial carcinomas of renal transplant patients, suggesting a potential aetiological role of polyoma virus in post-transplant bladder malignancies. However, they acknowledge that it is possible that tumour cells are more susceptible to BK virus infection than normal cells, and thus that the infection is a consequence rather than a cause. Still this hypothesis may warrant further investigation.

It is conceivable that our findings were because of chance, unmeasured confounding or other biases. Differential misclassification due to recall bias is possible, although we attempted to minimize this through the use of a pictorial guide of common medications. Moreover, it seems unlikely that bladder cancer patients would recognize oral glucocorticoids as a possible aetiological factor. Another advantage of our study was that both cases and controls were drawn from the general population; among cases, over 95% reported having a valid driver's license (those <65 years) or being enrolled in Medicare (those 65 years and older), ensuring our control group was represented within our case group. Our study population was relatively large, although the statistical power diminished in certain categories (i.e., by duration of use). We assessed the potential confounding effects of multiple factors and confounding by indication was unlikely, as we did not find systematic differences in the ORs for oral glucocorticoid use by reason for use. A weaker association observed with inhaled steroids is plausible because of the minimal systemic effects of inhaled steroids (Hardman *et al*, 1996; Zoorob and Cender, 1998). Oral glucocorticoid users appeared to be at an enhanced risk of specifically developing invasive, TP53 positive (3+ staining intensity) bladder cancer compared with non-users. This is supported by previous research that has observed more rapid progression of transitional cell carcinomas in immunosuppressed patients (Wang *et al*, 2002). This is perhaps because tumours are able to progress more quickly with diminished immunosurveillance. Nonetheless, our findings will need to be confirmed or refuted in future studies. If an enhanced risk of bladder cancer, particularly advanced disease is confirmed, it might indicate the need for closer monitoring of individuals who regularly take glucocorticoids.

## Figures and Tables

**Table 1:** Selected characteristics of bladder cancer cases and controls

	**Controls^a^**		**Cases^b^**	
	***n*=1083**	**(%)**	***n*=786**	**(%)**
*Age (years)*				
<55	263	(24)	155	(20)
55–63	259	(24)	210	(27)
64–68	235	(22)	172	(22)
⩾69	326	(30)	249	(32)
				
*Gender*				
Male	667	(61)	597	(76)
Female	416	(39)	189	(24)
				
*Smoking*				
Never	365	(33)	142	(18)
Former	538	(50)	388	(49)
Current	180	(17)	256	(33)
				
*Pack-years*				
⩽32	412	(60)	249	(39)
>32	275	(40)	386	(61)
				
*Education*				
High school or technical college	498	(47)	560	(72)
College	364	(33)	171	(22)
Graduate or professional school	216	(20)	48	(6)
				
*Family history of bladder cancer*				
No	1033	(99)	679	(95)
Yes	14	(1)	37	(5)
				
*Histology* c				
Transitional	—	—	676	(98)
Non-transitional	—	—	12	(2)
				
*Stage*				
Non-invasive; low grade	—	—	417	(63)
Non-invasive; high grade	—	—	52	(8)
Invasive	—	—	190	(29)
Carcinoma *in situ*	—	—	29	(4)

a Five controls missing data on education and 36 missing data on family history.

b Seven cases missing data on education, 70 missing data on family history, 98 missing data on transitional cell status and 98 missing data on bladder cancer stage.

c Non-transitional cell carcinomas include 2 spindle cell carcinoma, 3 small cell carcinoma, 1 squamous cell carcinoma *in situ*, 4 squamous cell carcinomas and 2 adenocarcinoma.

**Table 2:** Adjusted odds ratios (ORs) and confidence intervals (CIs) for oral glucocorticoid use among cases and controls

	**Controls^a^**	**All bladder cancers^b^**	
	***n*=1083**	***n*=786**	**Adjusted OR^c^ (95% CI)**
*Glucocorticoid use*			
No use	1032	725	1.00 (reference)
Both oral and inhaled	9	11	2.17 (0.87–5.42)
Oral only	42	50	1.78 (1.15–2.76)
Inhaled only	32	38	1.52 (0.92–2.51)
			
*Oral glucocorticoid use* d			
No	1032	725	1.00 (reference)
Yes	51	61	1.85 (1.24–2.76)
Former	33	35	1.50 (0.91–2.48)
Current	17	21	2.18 (1.11–4.28)
			
*Total duration of oral glucocorticoid use* d			
No use	1032	725	1.00 (reference)
⩽2 years	38	46	1.87 (1.18–2.96)
2–5 years	8	7	1.29 (0.50–3.64)
>5 years	4	8	3.39 (0.98–11.74)
			
*Reason for oral glucocorticoid use* e			
No use	1032	725	1.00 (reference)
Respiratory conditions and asthma	15	16	1.73 (0.83–3.60)
Musculoskeletal and connective tissue disease	20	21	1.40 (0.74–2.68)
Neoplasm	1	4	3.51 (0.39–31.90)
Allergy	3	9	7.39 (1.93–28.29)
Gastrointestinal disease	5	5	2.10 (0.57–7.66)
Other	10	3	0.46 (0.12–1.70)

a One control missing data on current/former oral glucocorticoid status and one control missing data on duration of oral glucocorticoid use.

b Five cases missing data on current/former oral glucocorticoid status.

c Model adjusted for age, sex and smoking status.

d Excludes 70 patients who took only inhaled steroids.

e Excludes 90 patients with any inhaled steroid use; reason for use may include multiple conditions or no conditions for each individual.

**Table 3:** Adjusted odds ratios (ORs) (95% confidence interval (CI)) for pathologically confirmed transitional cell (urothelial) carcinomas among users of oral glucocorticoids, stratified by histological type, disease stage and TP53 staining intensity

	**Oral glucocorticoid use^a^**				
	**No**		**Yes**		
	***n***	**%**	***n***	**%**	**Adjusted OR^b^ (95% CI)**
*Disease stage*					
Non-invasive; low grade	387	61%	30	56%	1.66 (1.03–2.70)
Non-invasive; high grade	48	8%	4	7%	2.03 (0.68–6.06)
Invasive	173	27%	17	31%	2.12 (1.17–3.85)
Carcinoma *in situ*	26	4%	3	6%	3.55 (0.98–12.85)
					
*p53 Staining intensity* c					
<3	440	73%	37	70%	1.79 (1.13–2.82)
3+	159	27%	16	30%	2.35 (1.26–4.36)

a Excludes 38 cases who reported taking only inhaled steroids.

b Model adjusted for age, sex and smoking status.

c 36 individuals missing data on p53 staining intensity.

## References

[bib1] Adami J, Gabel H, Lindelof B, Ekstrom K, Rydh B, Glimelius B, Ekbom A, Adami H-O, Granath F (2003) Cancer risk following organ transplantation: a nationwide cohort study in Sweden. Br J Cancer 89: 1221–1227 14520450 10.1038/sj.bjc.6601219 PMC2394311

[bib2] Asten P, Barrett J, Symmons D (1999) Risk of developing certain malignancies is related to duration of immunosuppressive drug exposure in patients with rheumatic diseases. J Rheumatol 26: 1705–1714 10451066

[bib3] Baker G, Kahl L, Zee B, Stolzer B, Agarwal A, Medsger T (1987) Malignancy following treatment of rheumatoid arthritis with cyclophosphamide: long-term case–control follow-up study. Am J Med 83: 1–9 10.1016/0002-9343(87)90490-6 3605161

[bib4] Birkeland S, Lokkegaard H, Storm H (2000) Cancer risk in patients on dialysis and after renal transplantation. Lancet 355: 1886–1887 10866449 10.1016/s0140-6736(00)02298-4

[bib5] Birkeland S, Storm H, Lamm L, Barlow L, Blohme I, Forsberg B, Eklund B, Fjeldborg O, Friedberg M, Frodin L, Glattre E, Halvorsen S, Holm N, Jakobsen A, Jorgensen H, Ladefoged J, Lindholm T, Lungren G, Pukkala E (1995) Cancer risk after renal transplantation in the Nordic countries, 1964–1986. Int J Cancer 60: 183–189 7829213 10.1002/ijc.2910600209

[bib6] Breslow N, Day N (1980) Statistical methods in cancer research. Volume I – The analysis of case-control studies. IARC Sci Publ 32: 338 7216345

[bib7] Buzzeo B, Heisey D, Messing E (1997) Bladder cancer in renal transplant recipients. Urol J 50: 525–528 10.1016/S0090-4295(97)00305-1 9338726

[bib8] Fortuny J, Kogevinas M, Zens MS, Schned A, Andrew AS, Heaney J, Kelsey KT, Karagas MR (2007) Analgesic and anti-inflammatory drug use and risk of bladder cancer: a population based case control study. BMC Urol 7: 13 17692123 10.1186/1471-2490-7-13 PMC2018698

[bib9] Hardman J, Gilman A, Limbird L (1996) Goodman & Gilman's The Pharmacological Basis of Therapeutics. Ninth edn. McGraw-Hill: New York

[bib10] Jensen A, Thomsen H, Engebjerg M, Olesen A, Friis S, Karagas M, Sorensen H (2009) Use of oral glucocorticoids and risk of skin cancer and non-Hodgkin's lymphoma: a population-based case–control study. Br J Cancer 100: 200–205 19034275 10.1038/sj.bjc.6604796 PMC2634665

[bib11] Johansson SL, Cohen SM (1997) Epidemiology and etiology of bladder cancer. Semin Surg Oncol 13: 291–298 9259084 10.1002/(sici)1098-2388(199709/10)13:5<291::aid-ssu2>3.0.co;2-8

[bib12] Kamal M, Soliman S, Shokeir A, Abol-Enein H, Ghoneim M (2007) Bladder carcinoma among live-donor renal transplant recipients: a single-centre experience and a review of the literature. BJU Int 101: 30–35 17850360 10.1111/j.1464-410X.2007.07210.x

[bib13] Karagas M, Cushing G, Greenberg E, Mott L, Spencer S, Nierenberg D (2001) Non-melanoma skin cancers and glucocorticoid therapy. Br J Cancer 85: 683–686 11531252 10.1054/bjoc.2001.1931 PMC2364134

[bib14] Kelsey K, Hirao T, Hirao S, Devi-Ashok T, Nelson H, Andrew A, Colt J, Baris D, Morris J, Schned A, Karagas M (2005) TP53 alterations and patterns of carcinogen exposure in a U.S. population-based study of bladder cancer. Int J Cancer 117: 370–375 15906354 10.1002/ijc.21195

[bib15] Kelsey K, Hirao T, Schned A, Hirao S, Devi-Ashok T, Nelson H, Andrew A, Karagas M (2004) A population-based study of immunohistochemical detection of p53 alteration in bladder cancer. Br J Cancer 90: 1572–1576 15083187 10.1038/sj.bjc.6601748 PMC2409723

[bib16] Kinlen L (1985) Incidence of cancer in rheumatoid arthritis and other disorders after immunosuppressive treatment. Am J Med 78: 44–49 10.1016/0002-9343(85)90245-1 3970040

[bib17] Knight A, Askling J, Granath F, Sparen P, Ekbom A (2004) Urinary bladder cancer in Wegener's granulomatosis: risks and relation to cyclophosphamide. Ann Rheum Dis 63: 1307–1311 15130900 10.1136/ard.2003.019125 PMC1754772

[bib18] Kyllonen L, Pukkala E, Eklund B (1994) Cancer incidence in a kidney-transplanted population. Transpl Int 7: S350–S352 11271249 10.1111/j.1432-2277.1994.tb01389.x

[bib19] Kyllonen L, Salmela K, Pukkala E (2000) Cancer incidence in a kidney-transplanted population. Transpl Int 13: S394–S398 11112040 10.1007/s001470050369

[bib20] Manfredi R, Sabbatani S, Calza L, Chiodo F (2006) Bladder carcinoma and HIV infection during the highly active antiretroviral therapy era: A rare, but intriguing association. Two case reports and literature review. Scand J Infect Dis 38: 566–570 16798716 10.1080/00365540500434653

[bib21] Master V, Meng M, Grossfeld G, Koppie T, Hirose R, Carroll P (2004) Treatment and outcome of invasive bladder cancer in patients after renal transplantation. J Urol 171: 1085–1088 14767276 10.1097/01.ju.0000110612.42382.0a

[bib22] Radis CD, Kahl LE, Baker GL, Wasko MC, Cash JM, Gallatin A, Stolzer BL, Agarwal AK, Medsger Jr TM, Kwoh CK (1995) Effects of cyclophosphamide on the development of malignancy and on long-term survival of patients with rheumatoid arthritis: a 20-year follow-up study. Arthritis Rheum 38: 1120–1127 7639809 10.1002/art.1780380815

[bib23] Roberts I, Besarani D, Mason P, Turner G, Friend P, Newton R (2008) Polyoma virus infection and urothelial carcinoma of the bladder following renal transplantation. Br J Cancer 99: 1383–1386 18971934 10.1038/sj.bjc.6604711 PMC2579688

[bib24] Schned A, Andrew A, Marsit C, Zens M, Kelsey K, Karagas M (2007) Survival following the diagnosis of noninvasive bladder cancer: WHO/International Society of Urological Pathology *vs* WHO Classification Systems. J Urol 178: 1196–1200 17698105 10.1016/j.juro.2007.05.126

[bib25] Scosyrev E, Noyes K, Feng C, Messing E (2009) Sex and racial differences in bladder cancer presentation and mortality in the US. Cancer 115: 68–74 19072984 10.1002/cncr.23986

[bib26] Setoguchi S, Solomon D, Weinblatt M, Katz J, Avorn J, Glynn R, Cook E, Carney G, Schneeweiss S (2006) Tumor necrosis factor alpha antagonist use and cancer in patients with rheumatoid arthritis. Arthritis Rheum 54: 2757–2764 16947774 10.1002/art.22056

[bib27] Silverman D, Devesa S, Moore L, Rothman N (2006) Bladder cancer. In Cancer Epidemiology And Prevention Schottenfeld D, JF Fraumeni J (eds). Oxford University Press: New York

[bib28] Sorensen H, Mellemkjaer L, Nielsen G, Baron J, Olsen J, Karagas M (2004) Skin cancers and non-Hodgkin lymphoma among users of systemic glucocorticoids: a population-based cohort study. J Natl Cancer Inst 96: 709–711 15126608 10.1093/jnci/djh118

[bib29] Talar-Williams C, Hijazi YM, Walther MM, Linehan WM, Hallahan CW, Lubensky I, Kerr GS, Hoffman GS, Fauci AS, Sneller MC(1996) Cyclophosphamide-induced cystitis and bladder cancer in patients with Wegener granulomatosis. Ann Intern Med 124: 477–484 8602705 10.7326/0003-4819-124-5-199603010-00003

[bib30] Travis LB, Curtis RE, Glimelius B, Holowaty EJ, Van Leeuwen FE, Lynch CF, Hagenbeek A, Stovall M, Banks PM, Adami J, Gospodarowicz MK, Wacholder S, Instip PD, Tucker MA, Boice Jr JD (1995) Bladder and kidney cancer following cyclophosphamide therapy for non-Hodgkin's lymphoma. J Natl Cancer Inst 87: 524–530 7707439 10.1093/jnci/87.7.524

[bib31] Vajdic C, McDonald S, McCredie M, Leeuwen Mv, Stewart J, Law M, Chapman J, Webster A, Kaldor J, Grulich A (2006) Cancer incidence before and after kidney transplantation. JAMA 296: 2823–2831 17179459 10.1001/jama.296.23.2823

[bib32] Volkmer B, Seidl-Schlick E, Bach D, Romics I, Kleinschmidt K (2005) Cyclophosphamide is contraindicated in patients with a history of transitional cell carcinoma. Clin Rheumatol 24: 319–323 16034647 10.1007/s10067-004-1032-2

[bib33] Wallace K, Kelsey K, Schned A, Morris J, Andrew A, Karagas M (2009) Selenium and risk of bladder cancer: a population-based case–control study. Cancer Prev Res 2: 70–73 10.1158/1940-6207.CAPR-08-0046 PMC2640833 19139020

[bib34] Wang H, Hsieh H, Chen Y, Chiang C, Cheng Y (2002) The outcome of post-transplant transitional cell carcinoma in 10 renal transplant recipients. Clin Transplant 16: 410–413 12437619 10.1034/j.1399-0012.2002.01152.x

[bib35] Wu M, Lian J, Yang C, Cheng C, Chen C, Lee M, Shu K, Tang M (2004) High cumulative incidence of urinary tract transitional cell carcinoma after kidney transplantation in Taiwan. Am J Kidney Dis 43: 1091–1097 15168390 10.1053/j.ajkd.2004.03.016

[bib36] Zoorob R, Cender D (1998) A different look at corticosteroids. Am Fam Physician 58: 443–450 9713398

